# Corticosteroid injections in the temporomandibular joint temporarily alleviate pain and improve function in rheumatoid arthritis

**DOI:** 10.1007/s10067-021-05860-y

**Published:** 2021-07-21

**Authors:** Johanna Margaretha Kroese, Sigvard Kopp, Frank Lobbezoo, Per Alstergren

**Affiliations:** 1 Department of Orofacial Pain and Dysfunction, Academic Centre for Dentistry Amsterdam (ACTA), University of Amsterdam and Vrije Universiteit Amsterdam, Gustav Mahlerlaan 3004, 1081 LA Amsterdam, The Netherlands; 2 Department of Periodontology, Academic Centre for Dentistry Amsterdam (ACTA), University of Amsterdam and Vrije Universiteit Amsterdam, Amsterdam, The Netherlands; 3 Department of Preventive Dentistry, Academic Centre for Dentistry Amsterdam (ACTA), University of Amsterdam and Vrije Universiteit Amsterdam, Amsterdam, The Netherlands; 4 Karolinska Institutet, Department of Dental Medicine, Section for Orofacial Pain and Jaw Function, Huddinge, Sweden; 5 Scandinavian Center for Orofacial Neurosciences (SCON), Malmö, Sweden; 6 Specialized Pain Rehabilitation, Skåne University Hospital, Lund, Sweden; 7 Faculty of Odontology, Orofacial Pain Unit, Malmö University, Malmö, Sweden

**Keywords:** Corticosteroid injection, Pain, Rheumatoid arthritis, Temporomandibular joint

## Abstract

**Objectives:**

To evaluate the effect of corticosteroid injections in the painful temporomandibular joint (TMJ) of patients with rheumatoid arthritis (RA) in relation to systemic inflammatory activity.

**Method:**

Examination of 35 patients (median age 54 years; 89% female) included maximum mouth opening capacity, degree of anterior open bite (AOB), TMJ pain intensity at rest, and crepitus. Serum levels of rheumatoid factor (RF), erythrocyte sedimentation rate (ESR), C-reactive protein (CRP), serotonin, and plasma levels of interleukine-1β (IL-1β) were determined. Out of the 70 examined joints, 53 joints received a corticosteroid (methylprednisolone) injection after the clinical examination at baseline (T0). The examination was repeated for all patients at T1 (median 3.1 weeks after T0), and for 21 patients at T2 (median 6.3 weeks after T1), of whom 20 patients received a second injection at T1.

**Results:**

Maximum mouth opening capacity significantly increased, and TMJ pain intensity significantly decreased between T0 and T1, but these improvements were no longer present at T2. No differences were found in AOB between the time points. Of the joints that received an injection at T0, 19 joints had pretreatment crepitus, which resolved in eight joints at T1. No correlations were found between the change in mouth opening capacity or TMJ pain intensity and ESR, CRP, serotonin, or IL-1β.

**Conclusions:**

Methylprednisolone injections in the TMJ alleviate pain and improve mouth opening capacity for approximately 3 weeks, allowing patients to perform jaw exercises during this timeframe of temporary relief. It thus seems useful for the short-term management of TMJ involvement in RA.

**Table:** 

**Key Points** • *In rheumatoid arthritis, corticosteroid injection in the temporomandibular joint alleviates pain and improves function.* • *The clinical improvement achieved with methylprednisolone injections lasts for approximately 3 weeks.* • *Corticosteroid injections could be used to facilitate and support additional noninvasive, conservative treatment options.*

## Introduction

Rheumatoid arthritis (RA) is an auto-immune disease affecting the synovial joints [1]. RA symptoms usually start bilaterally in small peripheral joints [2], but may involve other joints as well, including the temporomandibular joint (TMJ) [3]. When the TMJ is involved, pain usually occurs within 2 years following general disease symptom onset, while pain-related dysfunction and structural changes develop with time. Early recognition and treatment are thus recommended to minimize irreversible damage [4]. TMJ pain can also negatively influence the oral health-related quality of life [5], a subjective measure of disease burden, further supporting the need for treatment.

As recommended by the European League Against Rheumatism (EULAR), standard management of RA consists of systemic pharmacological treatment with disease-modifying antirheumatic drugs (DMARDs) [6]. Occasionally, corticosteroid injections are used to alleviate pain and reduce swelling in joints that do not or insufficiently respond to systemic treatment or experience a local flare-up. The most commonly injected joint is the knee, usually resulting in pain relief for approximately 8 weeks, but other joints can be injected as well [7].

In people with arthrogenous TMJ pain, corticosteroid injections were shown to be an effective method for pain reduction [8]. In patients with RA, a positive effect on function and subjective complaints was also found [9]. The clinical outcome of corticosteroid injections, in patients with several rheumatic diseases combined, was related to pretreatment synovial fluid concentrations of tumor necrosis factor-alpha [10] and serotonin [11], as well as pretreatment systemic concentrations of serotonin [11]. However, specific data on patients with RA is limited.

Therefore, the aim of the current study was to evaluate the clinical effect of corticosteroid injections in the painful TMJ of patients with RA, which were performed as a part of routine care, in relation to systemic inflammatory activity. We hypothesize improvement of pain and, consequently, improvement of function after treatment with corticosteroid injections. Furthermore, we hypothesize that lower pretreatment systemic inflammatory activity, as assessed by CRP and ESR, and lower pretreatment levels of serotonin and interleukin-1β result in better treatment effects on TMJ pain and function.

## Materials and methods

### Patients

A total of 35 patients, 31 woman and 4 men, with RA according to the 1987 classification criteria of the American College of Rheumatology [12], were included in this study. The patients were referred to the specialist clinic for Orofacial Pain and Jaw Function (Karolinska Institutet, Institution of Odontology, Department of Clinical Oral Physiology, Huddinge, Sweden) by rheumatologists in the Stockholm area, Sweden. The patients were included and examined between 1990 and 2006. Systemic pharmacological treatment of the general disease was provided by the referring rheumatologists; no specific data is available. The period of data collection was mostly before the introduction of biologics, which means that the medication profiles do not fully correspond to current guidelines. However, the aim of this study was to measure the effect of local treatment with corticosteroids in the TMJ. It may thus be considered as an advantage that the results are not influenced by the efficient general treatment effect of biologics, which can also affect the TMJ.

This project has a prospective cohort study design, and was approved by the regional ethical committee at Karolinska Institutet, Stockholm, Sweden (176/91; 310/97; 142/02; 03–2004) according to the Declaration of Helsinki. Written informed consent was obtained from all study participants.

### Assessment of subjective symptoms and clinical signs

The clinical examination included the assessment of several variables, further described below. All clinical examinations were performed by two experienced examiners (PA, SK), and the two examiners were calibrated regularly throughout the years, both theoretically and clinically, in order to prevent drift.

#### Variables on an individual level

The maximum voluntary mouth opening was measured in millimeters between the right central incisors, with the vertical overbite added.

The degree of anterior open bite (AOB) was assessed by recording of the occlusal contacts upon hard biting on a double occlusal foil in intercuspid position (2 × 8 µm, Occlusions-Prüf-Folie, GHM Hanel Medizinal, Nürtingen, Germany). Both the left side and the right side were assessed, and the following scores were used: 0 = occlusal contacts including the canine, 1 = no contacts anterior to the first premolar, 2 = no contacts anterior to the second premolar, 3 = no contacts anterior to the first molar, 4 = no contacts anterior to the second molar, and 5 = no occlusal contact. The sum of the scores of both sides was used in the analyses as an estimation of the degree of AOB. None of the patients in this study was edentulous, and the possible score thus ranged from 0 to 9. Score 9 (4 + 5) means that only one contact between two opposing posterior molars exists on one side.

#### Variables on a joint level

Local TMJ pain intensity at rest was assessed using either a 10-cm visual analogue scale (VAS; converted to a score of 0–10) or a numerical rating scale (NRS; 0–10) with the end points “no pain” (score 0) and “worst pain ever experienced” (score 10). Despite the use of two types of scales throughout the years of examinations, minimal influence on the results is expected due to the high correspondence between the two types of scales [13].

Besides maximum voluntary mouth opening, participants were asked to perform maximum protrusion and maximum laterotrusion to both sides. Crepitus was recorded as present if crepitus was palpable or audible during at least one of these movements.

Probable clinical arthritis was defined according to Alstergren et al. [14], where “probable TMJ arthritis” is considered present if a joint has the combination of pain on maximum mouth opening and a contralateral laterotrusion of less than 8 mm.

### Blood sampling

Venous blood was collected at the start of the first visit and immediately before the clinical examination, to determine serum levels of rheumatoid factor (RF), erythrocyte sedimentation rate (ESR), C-reactive protein (CRP), and serotonin, as well as plasma levels of interleukine-1β (IL-1β). Rheumatoid factor titers below 15 IE/mL and C-reactive protein levels below 10 mg/L were considered as zero values according to the standard procedures of the accredited laboratory at the Department of Clinical Chemistry at Karolinska University Hospital, Huddinge, Sweden.

### Treatment and examination schedule

After the clinical examination at the first visit (T0), painful TMJs received an injection with glucocorticoid methylprednisolone (40 mg/mL) with lidocaine (10 mg/mL) added (Depo-Medrol cum lidocaine; Pfizer AB, Täby, Sweden). A volume of 0.7–0.8 mL was injected in the upper joint compartment of the TMJ. All injections were administered by a dentist with a specialization in orofacial pain and dysfunction, several years of experience in the use of TMJ corticosteroid injections, and more than 2500 synovial fluid samplings (SK or PA), without the use of imaging guidance. Participants also received individualized care with self-care instructions, including jaw exercises.

For all patients, the clinical examination was repeated (T1) after a median (interquartile range; IQR) interval of 3.1 (2.1–9.0) weeks. Based on the clinicians’ decision on desired follow-up within the clinical care setting, for 21 patients, the clinical examination was repeated again (T2) after a median (IQR) interval of 6.3 (4.3–17.9) weeks between T1 and T2.

### Statistical analyses

Non-parametric statistics were used throughout the study due to the characteristics of most measured variables. For descriptive statistics, median values and 25^th^/75^th^ percentiles are presented. Differences in maximum voluntary mouth opening, anterior open bite, and pain intensity between time points were tested with the Wilcoxon signed-ranks test. To assess the correlation between clinical effect of the corticosteroid injections and systemic variables, the changes in maximum voluntary mouth opening and pain intensity between T0 and T1 were used as the clinical variables. The significance of correlations was tested with the Spearman’s ranked correlation test. A probability level of *p* < 0.05 was considered as significant.

## Results

Data were collected for 70 joints in 35 RA patients. Table 1 shows the characteristics of the study sample. During the first visit (T0), all patients received a corticosteroid injection in one or both joints; in total, 53 out of 70 joints were injected. All patients had a second visit (T1), and 21 patients also had a third visit (T2), out of whom 20 patients received another TMJ injection during the visit at T1.

**Table 1:** Characteristics of the study sample

		Percentile		
	Median	25^th^	75^th^	n
Age, years	54	38	62	35
Gender (M/F)				4/31
Duration of general joint symptoms (years	8	4	20	34
Time between onset of general and TMJ symptoms (years)	4	1	15	33
RF positivity, *n* (%)				22 (63)
Erythrocyte sedimentation rate	27	18	42	30
C-reactive protein	11	0.01	28	33
Thrombocyte particle count	335	274	420	32
Time between T0 and T1 (weeks)	3.1	2.1	9	35
Time between T1 and T2 (weeks)	6.3	4.3	17.9	21

*n* number of observations, *TMJ* temporomandibular joint, *RF* rheumatoid factor

### Maximum mouth opening capacity

**Fig. 1:**
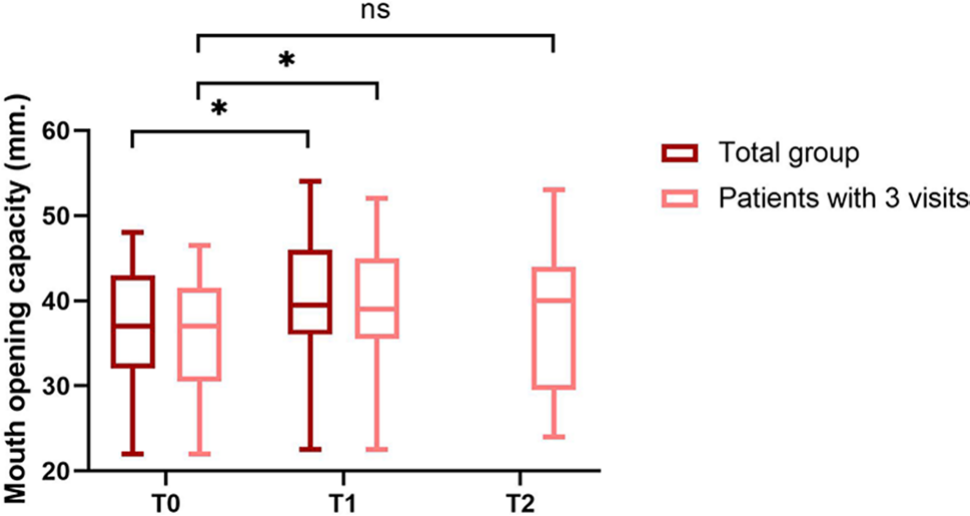
Maximum mouth opening capacity in 35 patients with rheumatoid arthritis. All patients received a corticosteroid injection in one or both temporomandibular joints (TMJs) at T0, of whom 21 patients had a third visit at T2. The median (IQR) interval was 3.1 (2.1–9.0) weeks between T0 and T1, and 6.3 (4.3–17.9) weeks between T1 and T2. A significant result (*p* < 0.05) is indicated by an asterisk, while “ns” indicates no statistically significant difference

The median maximum mouth opening capacity significantly increased between T0 and T1 (from 37 to 40 mm, *p* = 0.004). For the 21 patients that had a third visit, the median maximum mouth opening capacity slightly increased between T1 and T2, but this change was not significant (from 39 to 40 mm, *p* = 0.139), and there also was no significant difference between T0 and T2 (*p* = 0.432; Fig. 1).

### Anterior open bite

The AOB did not differ significantly between T0 and T1 (median 0 and 1, respectively, *p* = 0.307), and also not between T1 and T2 (median 1 and 0, respectively, *p* = 0.109), nor between T0 and T2 (median 0 for both, *p* = 0.478) for patients that had three visits.

### Temporomandibular joint pain intensity

**Fig. 2:**
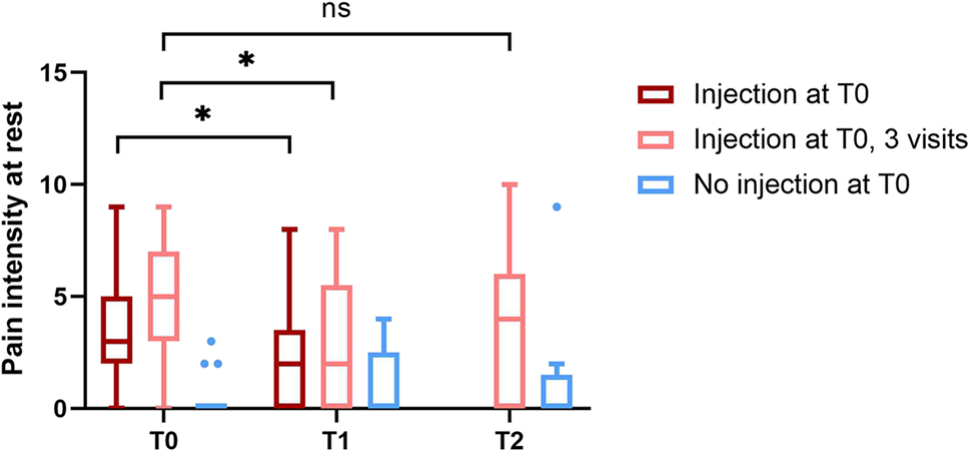
Pain intensity at rest in the temporomandibular joint (TMJ) of patients with rheumatoid arthritis. Forty-seven TMJs received a corticosteroid injection at T0, of which for 29 TMJs, data is available during a third visit at T2, while 17 TMJs in the same patient group did not receive corticosteroid injections at T0. The median (IQR) interval was 3.1 (2.1–9.0) weeks between T0 and T1, and 6.3 (4.3–17.9) weeks between T1 and T2. A significant result (*p* < 0.05) is indicated by an asterisk, while “ns” indicates no statistically significant difference

Results are presented in Fig. 2. The TMJ-pain intensity at rest significantly decreased between T0 and T1 (median 3 and 2, respectively, *p* = 0.001) for the joints that received an injection at T0. For patients that had a third visit, no further difference was found between T1 and T2 (median 2 and 4, respectively, *p* = 0.123), nor between T0 and T2 (median 5 and 4, respectively, *p* = 0.228). For the joints that did not receive an injection at T0, no differences in TMJ-pain intensity over time were found (median 0 at all time points, Fig. 2).

### Crepitus

**Fig. 3:**
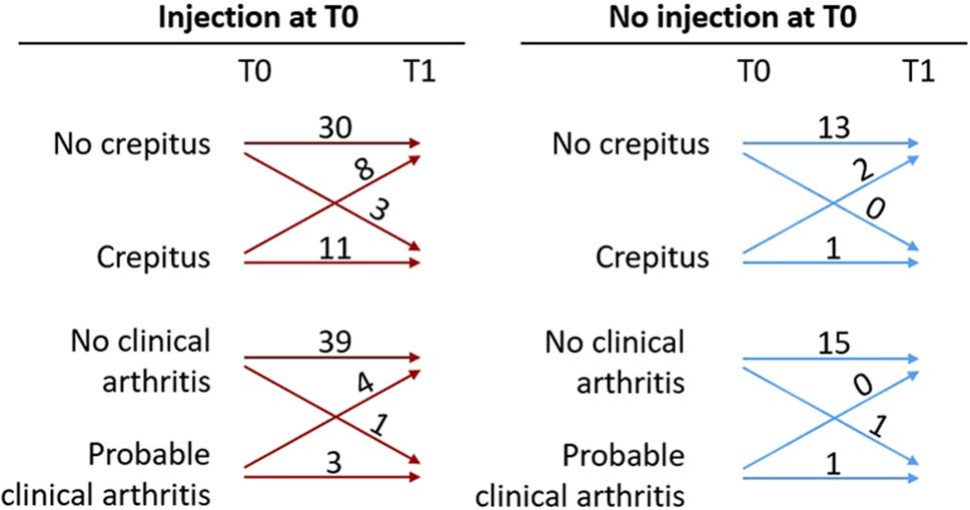
Crepitus and fulfillment of the clinical criteria for arthritis in the temporomandibular joint of patients with rheumatoid arthritis. Numbers of transitions between T0 and T1 from crepitus to no crepitus and vice versa (in 68 joints, of which 52 joints received a corticosteroid injection at T0), and from probable clinical arthritis to no clinical arthritis and vice versa (in 64 joints, of which 47 joints received a corticosteroid injection at T0). The median (IQR) interval was 3.1 (2.1–9.0) weeks between T0 and T1

Data on crepitus were available for 68 joints. Figure 3 shows the transitions from crepitus to no crepitus and vice versa between T0 and T1, for 52 joints that received a corticosteroid injection at T0, and 16 joints that did not receive a corticosteroid injection at T0. At T2, data are available for 34 joints that received an injection at T0, of which eleven joints had crepitus—two new cases—and nine joints that already had crepitus at T0 and/or T1. Of the joints that did not receive an injection at T0, only one joint had crepitus at T1. This joint received a corticosteroid injection at T1 and was then crepitus-free at T2.

### Clinical arthritis

Data on probable clinical arthritis is available for 64 joints. Figure 3 shows the transition from probable clinical arthritis to no clinical arthritis and vice versa between T0 and T1, for 47 joints that received a corticosteroid injection at T0, and 17 joints that did not receive a corticosteroid injection at T0.

### Relation with pretreatment systemic variables

The change in maximum voluntary mouth opening between T0 and T1 did not correlate to pretreatment serum levels of ESR (*r*_s_ =  − 0.057, *n* = 30, *p* = 0.766), CRP (*r*_s_ =  − 0.221, *n* = 33, *p* = 0.215), and serotonin (*r*_s_ =  − 0.273, *n* = 28, *p* = 0.160), nor to plasma levels of IL-1β (*r*_s_ =  − 0.257, *n* = 27, *p* = 0.196).

The change in pain intensity between T0 and T1 also did not correlate to pretreatment serum levels of ESR (*r*_s_ = 0.087, *n* = 36, *p* = 0.613), CRP (*r*_s_ = 0.202, *n* = 40, *p* = 0.212), and serotonin (*r*_s_ = 0.213, *n* = 36, *p* = 0.213), nor to plasma levels of IL-1β (*r*_s_ =  − 0.238, *n* = 31, *p* = 0.196).

## Discussion

This study indicates that methylprednisolone injections in a painful TMJ of patients with RA alleviate symptoms and improve function for approximately 3 weeks. Although all injected joints were painful, most joints did not fulfill the novel diagnostic criteria for “probable clinical TMJ arthritis.” This study could not establish that pretreatment systemic inflammatory activity is related to the treatment effect.

The methylprednisolone used for TMJ injection in this study is a crystalized corticosteroid with small- to medium-sized crystals that are known to retain in the tissues with pharmacological effects for 1 to 3 weeks after injection [15], which corresponds to our results. However, while injection in the RA knee joint results in a duration of remission for approximately 8 weeks, and decrease in joint effusion can even last up to 1 year [7], the current results show that both pain and dysfunction in the TMJ revert to post-treatment levels relatively quickly after the injection. This corresponds to the short- to medium-term effects of intra-articular injection with corticosteroids found in other populations with temporomandibular dysfunction (TMD) [16]. To attain long-term improvement of TMJ symptoms and function, addition of other treatment modalities is crucial. Despite the short duration, the combination of decrease in pain intensity and increase in maximum mouth opening achieved by corticosteroid injection does offer patients a temporary relief, but also allows them to perform jaw exercises without being hindered by pain. Injections could thus be used to facilitate additional noninvasive, conservative treatment of TMD.

As with any treatment, possible negative effects should always be considered when deciding on a treatment strategy, especially since corticosteroids have potent effects on most cell types. In case of corticosteroid injections in the hip and knee of osteoarthritis (OA) patients, adverse joint findings such as accelerated OA progression have been observed [17]. However, a study on corticosteroid injections in several types of joints—both large and small—of RA patients that included evaluation of possible negative effects showed a good tolerance and no serious adverse events, suggesting that the positive results outweigh the possible negative consequences in this patient group [18]. Furthermore, none of the studies that have investigated side effects such as accelerated bone tissue destruction have taken into account the intra-articular inflammatory activity. Since arthritis can cause pain as well as cartilage and bone tissue destruction, these effects may not be related to the treatment with corticosteroids. Possible side effects like skin atrophy in case of subcutaneous deposition and transient pain due to crystal-induced synovitis are well-documented adverse effects of corticosteroid injections. To minimize the risk of negative consequences, it is prudent to have the injections administered by experienced clinicians, which was the case within this study. However, no imaging guidance was used during the procedure. Several studies demonstrate increased accuracy of the injection procedure with ultrasound guidance [19], although efficacy of the ultrasound-guided injections seems similar to palpation-guided injections, as demonstrated in several joints of RA patients [20]. We thus expect no relevant influence of the lack of imaging guidance on the clinical results of this study.

In this study, the change in maximum mouth opening and pain intensity was not related to pretreatment systemic inflammatory activity. However, in a population of patients with various rheumatic diseases, Fredriksson et al. [11] found an association between treatment effect and pretreatment systemic levels of serotonin. Our finding is also in contrast with earlier findings of Alstergren et al. in seropositive RA patients, where TMJ pain on mandibular movement was correlated to systemic factors [21]. The current results do not confirm this association for RA patients, indicating that more research into how systemic inflammatory activity affects symptoms of TMJ arthritis is needed. In further research aimed at phenotyping patients that benefit from specific treatment modalities, preferably both systemic and local inflammatory mediators should be taken into account. The study by Fredriksson et al. shows that treatment effect of intra-articular corticosteroid injections was also associated with pretreatment levels of serotonin in local TMJ synovial fluid [11]. However, it was not possible to take this factor into account while analyzing the current results, because complete data on synovial fluid were not available.

No significant changes in the degree of anterior open bite (AOB) were found over time. AOB can be used as a coarse clinical sign of tissue destruction, and may develop over time in RA patients with TMJ involvement [4]. The duration of the current study was most likely too short to be able to observe a possible change in AOB, at least to be able to detect normalization due to a possible arrested bone tissue loss by the treatment and subsequent normalization of occlusion. A longer follow-up period would be necessary to investigate whether TMJ tissue destruction can be prevented by using corticosteroid injections. A study by Vallon et al. [22] with a 12-year follow-up did show a positive long-term result of TMJ corticosteroid injections in patients with RA on radiological signs of structural bone changes. However, the drop-out rate was high—only 12 out of the original 41 participants were examined clinically and radiographically after 12 years—and systemic treatments were not taken into account. Their results must therefore be interpreted with caution.

The limitations of the current study that may influence the generalizability of the conclusions, further discussed below, include the lack of a control group, the lack of information on individualized care regarding the TMJ, the use of the 1987 classification criteria for RA, the lack of detailed information on systemic pharmacological treatment, and that the medication profiles presumably do not fully correspond to current guidelines.

In studies that measure the effect of a certain treatment, it is preferable to have a non-treatment control group. On the other hand, one of the main aims of this study was to relate treatment effects to systemic inflammatory activity. In the current study, 17 joints did not receive treatment with a corticosteroid injection and showed no change in pain intensity over time. However, these joints cannot be considered as pure controls because the decision to inject was based on baseline pain intensity. In these joints, pain intensity was very low to absent throughout the duration of the study, and they are thus difficult to compare to the joints that did receive an injection. In addition, anti-inflammatory treatment in one joint may influence the contralateral joint through systemic or central mechanisms.

In addition to the corticosteroid injections, participants within this study received individualized care with self-care instructions, including jaw exercises. However, specific information was not available to take into account during the analysis of the results. Furthermore, the clinical relevance of the decrease in pain intensity and increase in maximum mouth opening capacity were not measured on a subjective level. In future research, the combination of corticosteroid injections and other treatment modalities, and the subjective effect of the injections—for example, as measured by possible changes in oral health-related quality of life—deserve further attention.

Since patients were included between 1990 and 2006, RA was diagnosed according to the 1987 classification criteria of the American College of Rheumatology (ACR). In 2010, the ACR and the European League Against Rheumatism (EULAR) published revised classification criteria [23]. These 2010 ACR/EULAR criteria put more emphasis on RA characteristics that emerge early in the disease course, in order to identify and treat RA patients earlier. However, the aim of this study was to evaluate the effect of TMJ corticosteroid injections in RA patients in general, regardless of their general disease duration. With a median duration of general joint symptoms of 8 years, we do not expect a significant influence of the 1987 classification criteria on the selected patient group. It does mean that patients possibly received a different pharmacological treatment, and at a later stage than they would have nowadays, as further discussed below.

Specific data on the pharmacological treatment of patients were not available and could thus not be analyzed as a possible confounding factor. The medication profiles presumably do not fully correspond to current guidelines, since the period of data collection was mostly before the introduction of biologics, which means that results can only be generalized to RA patients that are not on biologic therapy. However, it may also be considered an advantage as previously mentioned. Furthermore, although developments in the treatment of RA have substantially changed the course of the disease, nowadays frequently resulting in remission [24, 25], recent studies still report a high prevalence of TMJ involvement in RA [26, 27]. In patients with early RA, systemic pharmacological treatment seemed to have a positive effect on TMJ involvement for some, but not all patients [28, 29]. This corresponds to the course of RA disease in general, where individual joints can display persistent complaints or temporary flare-ups, resulting in the continuous use of corticosteroid injections in clinical practice. An interesting development in research to monitor in this context is the focus on injection with anti-TNF agents as an alternative for glucocorticoids [30, 31]. Altogether, this suggests that despite the greatly improved treatment modalities, targeted treatment for the TMJ remains necessary for a number of RA patients.

## Conclusion and implications

This study indicates that corticosteroid injections with methylprednisolone in the TMJ alleviate pain and improve mouth opening capacity for approximately 3 weeks. The temporary relief achieved with an injection can facilitate patients to perform jaw exercises, without being limited by pain. Corticosteroid injections thus seem useful for the short-term management of TMJ involvement in RA.

## Data Availability

Data are available on reasonable request to the corresponding author (JMK; j.m.kroese@acta.nl).
